# Femtosecond Laser Written Depressed-Cladding Waveguide 2 × 2, 1 × 2 and 3 × 3 Directional Couplers in Tm^3+^:YAG Crystal

**DOI:** 10.3390/mi11010001

**Published:** 2019-12-18

**Authors:** Nikolay Skryabin, Alexander Kalinkin, Ivan Dyakonov, Sergei Kulik

**Affiliations:** 1 Quantum Technologies Center and Faculty of Physics, Lomonosov Moscow State University, Leninskie gory 1, building 35, Moscow 119991, Russia; nikolay.skryabin@phystech.edu (N.S.); kalinkin@physics.msu.ru (A.K.); sergei.kulik@physics.msu.ru (S.K.); 2 Moscow Institute Physics and Technology, Institutskiy per. 9, Dolgoprudny 141701, Russia

**Keywords:** femtosecond laser writing, depressed-cladding waveguide, directional coupler, yttrium aluminum garnet, crystal, integrated photonics, quantum memory

## Abstract

Ion-doped crystal-based compact devices capable of beam splitting and coupling are enthralling for a broad range of classical and quantum integrated photonics applications. In this work, we report on the fabrication of depressed-cladding waveguide 2D 2 × 2, 1 × 2 and 3D 3 × 3 directional couplers in Tm${}^{3+}$:YAG crystal by femtosecond laser writing. The performances of the couplers are characterized at 810 nm, showing single-mode guidance, polarization independence, finely matched splitting ratios. These results open up new opportunities in the beneficial fabrication of 3D circuits and devices in crystals.

## 1. Introduction

A single platform integration of all the necessary components of the primary elemental base of optics and photonics faces difficulties similar to those encountered by the electronics industry in the 1950s. To obtain the required stability and scalability, integrated optical circuits must be placed into a monolithic chip. The search for such a solid-state platform for integrated photonics devices, where all necessary operations can be performed, is a key issue in this area.

Femtosecond laser writing is a technology for direct and permanent refractive index modification of a transparent dielectric material under the action of tightly focused ultrashort laser pulses. Moving the material relative to the focus, the elongated tracks are written inside the sample. The method enables the rapid fabrication of various kinds of three-dimensional integrated photonic elements and devices in a wide range of materials, such as optical glasses, crystals, ceramics, diamonds, and polymers [1]. Femtosecond laser written waveguides were firstly demonstrated in glasses by the Hirao group in 1996 [2]. Then in 2000, by analogy with glasses, waveguides writing was demonstrated inside crystalline quartz [3]. In general tracks written in glasses exhibit positive refractive index change whereas tracks in crystals exhibit a negative index modification. Refractive index modification (Δn) and form of the written tracks can be different depending on the material, laser parameters and focusing conditions. According to this, femtosecond laser written waveguides are classified into three basic types [4]: Type-I—a directly written waveguide, where written track has positive refractive index changed region relative to the unmodified volume of the sample, so this region acts as a waveguiding core; Type-II—a stress-induced waveguide, where the region with positive Δn is formed between two written tracks with negative Δn due to stress-induced effects; Type-III—a depressed cladding waveguide, where waveguide’s cladding consist of a number of tracks with negative Δn. Various kinds of femtosecond laser written waveguides have been of great interest for integrated photonics applications [1,4], including quantum computing [5,6] and quantum memory [7,8].

Quantum memory is an important component for quantum communications, so it is worth to pay attention to systems that have the potential to create solid-state quantum storage devices. To date, the only solid-state systems with large coherence time necessary for the implementation of quantum repeaters are rare-earth ion-doped crystals [9,10]. Since a photon echo-based quantum memory cell is an essential component of a quantum repeater [11], it is necessary to develop a waveguide manufacturing procedure in rare-earth ion-doped crystals and combine it with an integrated quantum communication platform. A variety of materials have been proposed as a platform for quantum integrated circuits, including rare-earth ion-doped crystals such as lithium niobate [12,13,14], YSO [7,8,15], fluorides [16,17], and KTP [14], but the complete integrated quantum-photon device with all the necessary components for a specific operation has not yet been demonstrated.

A Tm${}^{3+}$:YAG crystal is experimentally shown to be an excellent candidate for quantum storage in bulk optics [18,19,20]. In this study we focus on waveguide writing and integrated device fabrication in Tm${}^{3+}$:YAG crystal for quantum memory application. All three types of femtosecond laser written waveguides were demonstrated in both YAG single crystals and polycrystalline ceramics. Type-I waveguides were fabricated only under certain conditions in Cr:YAG crystal [21] and in Er/Ho:YAG ceramics [22]. Type-II waveguides have been realized in various ion-doped YAG crystals [23,24,25,26,27,28,29,30] and ceramics [31,32,33,34,35], but these waveguides supported only TM-polarized eigenmode [23,32,33]. Type-III waveguides were fabricated in various ion-doped YAG crystals [36,37,38,39,40,41] and ceramics [42,43,44,45,46,47,48,49]. Such waveguides support two orthogonally polarized eigenmodes [38,42]. Recently, an alternative waveguide cladding fabrication technique based on hexagonal microstructured lattices of laser written tracks has been demonstrated in Nd:YAG crystal [50].

Type-I waveguides suit best for implementation of complex integrated photonic devices: two-dimensional (2D) 1 × 2 beam splitter in LiNbO${}_{3}$ [51] and three-dimensional (3D) 1 × 4 beam splitters in BGO [52] and in LiNbO${}_{3}$ [53] were demonstrated. Fabrication of such elements with Type-II and Type-III waveguides faces substantial challenges: the design of the interaction region, waveguide cladding alignment, finding the preferential shape of the waveguide cladding, etc. The 2D 1 × 2 Y-splitters with square shape depressed cladding waveguides were realized in LiTaO${}_{3}$ [54], in Nd:YAG [55,56] and in sapphire [57]. The circularly shaped depressed cladding waveguides in Nd:YAG [58] and LiNbO${}_{3}$ [59] were adopted to inscribing 2D 1 × 2 Y-splitter and 3D 1 × 2 Y-splitter respectively. The lattice microstructured cladding waveguide 3D 1 × 3 beam splitter in LiNbO${}_{3}$ [60] and 1 × 4 beam splitter in KTP [61] were demonstrated. Recently, conventional 2 × 2 reconfigurable weakly coupled directional beamsplitter in LiNbO${}_{3}$ was demonstrated [62].

In this paper we experimentally demonstrate depressed-cladding waveguide-based 2D 2 × 2, 1 × 2 and 3D 3 × 3 directional couplers in Tm${}^{3+}$:YAG crystal. The coupling region is formed by removing several tracks from each of the waveguides. The fabricated waveguides show single-mode guiding in both orthogonal TM- and TE-polarizations at 810 nm.

## 2. Materials and Methods

**Figure 1:**
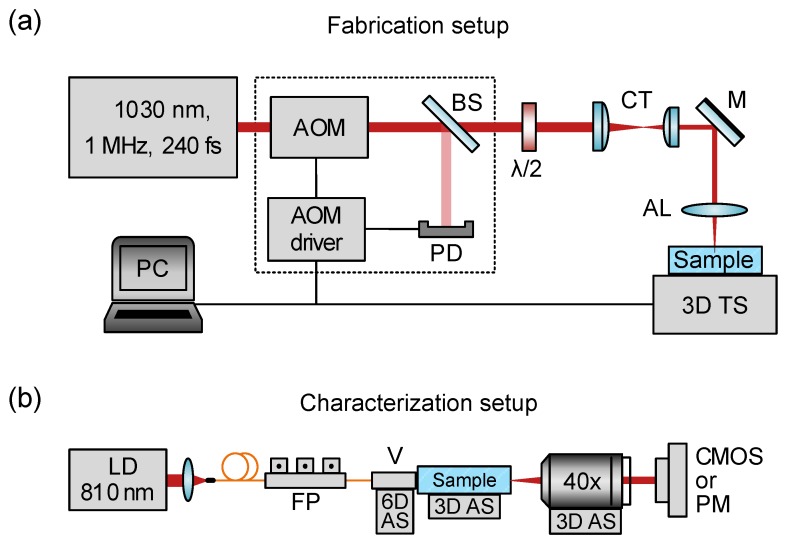
(**a**) The experimental setup for femtosecond laser writing. AOM—acousto-optic modulator, λ/2—half-wave plate, BS—beamsplitter, PD—photodiode, CT—cylindrical telescope, M—mirror, AL—aspheric lens (NA=0.55), 3D TS – automated three-axis translation stage. (**b**) Setup for characterization of integrated photonic elements. LD—laser diode, FP—fiber polarizer, V—v-groove fiber holder, 6D AS—six-axis mechanical alignment stage, 3D AS—three-axis mechanical alignment stage, 40X—optical objective, CMOS—beam profiler, PM—power meter.

The experimental setup for a femtosecond laser writing is shown in Figure 1a. Yb-doped fiber laser (Antaus, Avesta Project Ltd., Moscow, Russia) emits 240 fs pulses at 1 MHz repetition rate and 1030 nm wavelength. The power of laser radiation was locked to a desired value using an acousto–optical modulator (AOM) driven by an active feedback circuit. A small portion ≈2% of laser power is diverted from the main optical scheme and is continuously monitored by a photodiode PD. The photodiode signal and the reference voltage defining the required laser power level are fed into the AOM driver which adjusts the output laser power accordingly. We used a focal spot intensity distribution astigmatic correction to reduce the ellipticity and increase the uniformity of the written tracks [63,64]. The correction is introduced by the 10:3 cylindrical telescope (CT, a system of two cylindrical lenses with focal length values 200 mm and 60 mm). Laser pulses were focused inside the Tm${}^{3+}$:YAG sample (19 mm × 9 mm × 2 mm) using an aspheric lens (AL) with 0.55 numerical aperture (C230TMD-B, Thorlabs GmbH, Munich, Germany) to the 250 μm depth below the surface. The sample is mounted onto a three-axis positioner (FiberGlide3D, Aerotech Inc., Pittsburgh, PA, USA) and translated relative to the writing laser focal spot with a constant velocity of 1 mm/s. The polarization of the writing beam is set linear by a half-wave plate λ/2 along waveguide axis.

The fabricated structures were inspected with the bright field Axio Scope A1 optical microscope using 10X and 40X aberration-corrected optical objectives. The optical properties of the integrated photonic elements are characterized in the setup illustrated in Figure 1b. The 810 nm laser radiation is coupled to a single-mode waveguide connected to the v-groove fiber array mounted onto a v-groove holder on the six-axis mechanical alignment stage. The input polarization state is defined by a fiber polarization controller. The near-field spatial profile of the waveguide eigenmode is measured by imaging the output facet of the sample onto the CMOS camera (Beamage-4M, Gentec-EO Inc., Quebec City, QC, Canada) with 40X, NA=0.65 optical objective (RMS40X, Olympus Corporation, Tokyo, Japan). The far-field profile is imaged in the collimated configuration. The loss measurements are conducted using the power meter (PM100D, Thorlabs GmbH, Munich, Germany). Total losses (TL) are evaluated as a ratio between measured output or sum of output (after 40X optical objective) and input (after v-groove fiber holder before entering the chip) powers as $TL=-10\times log(P_{out}/P_{in})$, meanwhile, losses in the objective (OL) were also taken into account as an additional factor which was also experimentally measured in the same manner, so insertion loss (IL) is defined as IL=TL−OL. It should be noted that insertion loss contains propagation loss (PL), coupling loss (CL) and bending loss (BL), so IL=PL+CL+BL. Splitting ratio is defined as a normalized relation between output powers. Because of slight differences were observed in insertion losses and splitting ratios depending on the waveguide used for input, these values were averaged over all the input channels.

## 3. Results

### 3.1. Depressed-Cladding Waveguide

**Figure 2:**
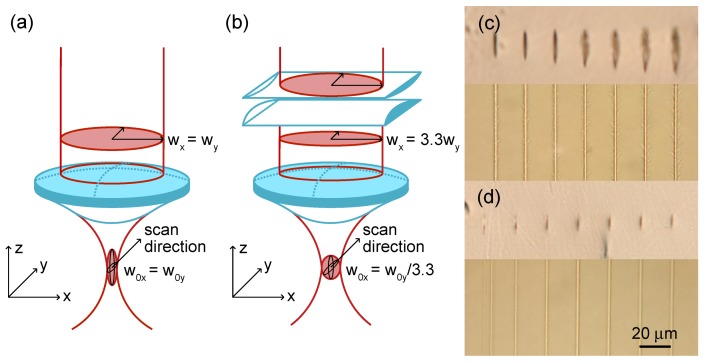
The illustration of beam focusing without (**a**) and with (**b**) the cylindrical telescope. The effect of the astigmatic beam shaping on the laser written tracks in Tm${}^{3+}$:YAG crystal: (**c**) cross-section and top views of the tracks without the cylindrical telescope applying 600 to 1200 nJ pulse energies, (**d**) cross-section and top views of the tracks created with the cylindrical telescope installed and pulse energies applied between 300–600 nJ.

The depressed cladding waveguide is the complex structure comprised of a series of precisely positioned tracks. The quality of the depressed cladding waveguide primarily depends on the quality of the single tracks forming the cladding structure. The central task for a depressed-cladding waveguide writing is locating the single track writing regime selection in the multiparameter space of the sample exposure characteristics. The ideal track should have an aspect ratio ≈1 and high smoothness for a cladding symmetry and low propagation losses, respectively. Therefore, instead of a symmetrical Gaussian writing beam (see Figure 2a), we use astigmatic beam shaping technique [63,64] to form the desired focal volume shape inside the sample and hence transform the track cross-section profile. The laser beam is shrinked 3.3 times along the Y axis with a 10:3 cylindrical telescope. The waist of the focal spot was increased by 3.3 times and the Rayleigh length reduced by 3.3 times (see Figure 2b). Modified tracks written in the YAG crystal sample without the cylindrical telescope are elongated along the writing beam direction and inhomogeneous. The cross-section and top view of the single structures created in Tm${}^{3+}$:YAG with pulse energies ranging from 600 to 1200 nJ are illustrated in Figure 2c. Even the track written with the lowest energy has highly asymmetric 18μm×2μm profile. The cylindrical telescope allows to minimize the writing energy and get rid of the unwanted track defects and achieve reduction of the track cross-section aspect ratio (see Figure 2d).

**Figure 3:**
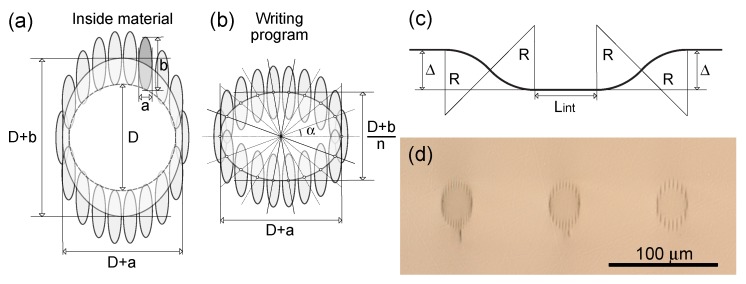
(**a**) Shape of the waveguide written inside the bulk of the sample crystal. (**b**) The writing program design guidelines to achieve the circular core depressed cladding waveguide. (**c**) The geometry of the s-shaped waveguide. (**d**) The cross-sections of the waveguides written with different number of tracks and fixed core diameter *D* = 28 μm (from left to right: *K* = 27, *K* = 22, *K* = 17).

The track written with 450 nJ pulse energy and cross-section size a=2μm and b=8μm was selected to form the depressed cladding waveguide. Then we have written a number of waveguides with different core diameters *D* (from 12 to 32 μm with 4 μm step) and cladding density ρ=D/K (defined as the core diameter per the number of tracks *K* forming the cladding). In order to fabricate the waveguides with diameters exactly equal to D inside material (see Figure 3a), the cladding writing program accounts for refraction of the focused beam inside the sample. The centers of the single tracks in the cladding are located on the ellipse with D+a and (D+b)/n axes spaced with the α=2π/K angle step (see Figure 3b). We have chosen to write the s-shaped waveguides (see Figure 3c) in order to select the ones which support light propagation with tolerable level of additional loss in the bent region. The s-shaped waveguide includes two bent regions consisting of circular bent waveguides with the bending radius *R*. The $L_{int}$ parameter is further used to set the interaction length of the directional couplers. The spacing between the track in the interaction region and input/output regions is set by parameter Δ = 125 μm. The cross-sections of the waveguides written with different number of tracks and fixed core diameter *D* = 28 μm (from left to right: *K* = 27, *K* = 22, *K* = 17) are shown in Figure 3d. Some of the fabricated waveguides had a crack underneath the cladding which however had no effect on the light propagation through the waveguides.

**Figure 4:**
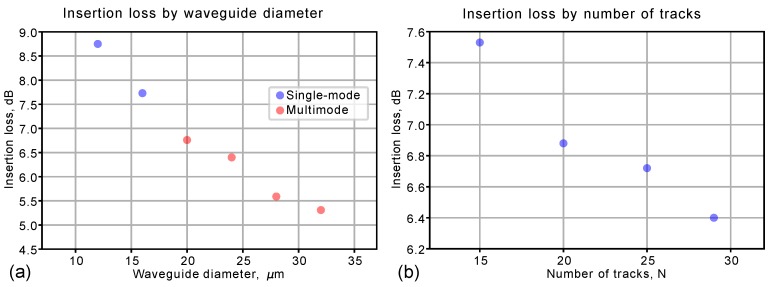
(**a**) The insertion loss measured for waveguides with different diameter. The number of waveguide varied but the density of the cladding is kept fixed ρ≈0.86μm for all structures. (**b**) The dependence of the insertion loss on the number tracks composing the cladding with the fixed diameter D=24μm.

The insertion loss measurement in both orthogonal TM- and TE-polarizations at 810 nm revealed that the waveguides are actually polarization independent. With increasing the diameter of the waveguide core the insertion loss fell and the waveguides became multimode (see Figure 4a). Also the insertion loss decreased with increased cladding density (see Figure 4b), due to reducing the leakage loss. The trade-off between the diameter defining the mode structure of the waveguide and the cladding density defines the optimal design of the depressed cladding waveguide. In this work we select D=16μm diameter waveguide which cladding consists of K=18 waveguides (the density of the cladding is ρ=0.89μm). This geometry was used for all further experiments with complex integrated structures.

### 3.2. Functional Integrated Photonic Elements

#### 3.2.1. The 2 × 2 and 1 × 2 Directional Couplers

**Figure 5:**
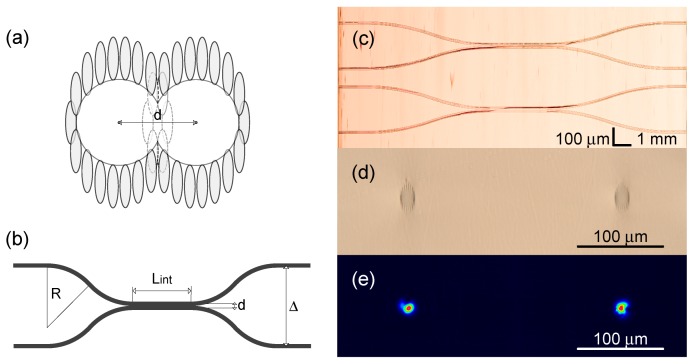
(**a**) The layout of the interaction region with several cladding tracks skipped. (**b**) The geometry of the 2 × 2 waveguide coupler. (**c**) The microscope image of the fabricated directional coupler structure. The image is stitched from a series of photographs with a smaller field of view. (**d**) The image of the output facet of the directional coupler. (**e**) The intensity profile on the output of the directional coupler with 48:52 splitting ratio.

The 2 × 2 directional coupler includes two waveguides brought at a close distance in the interaction region. The length of the interaction region and the distance between waveguide cores define the splitting ratio at the output of the coupler. The geometry of the directional coupler and the structure of the interaction region are presented in Figure 5a,b. The overall length of the directional coupler is 19 mm. The distance between the waveguides at the input and output facets is set to Δ= 250 μm to match the distance between the input optical fibers glued into a v-groove array with 250 μm groove spacing. The curvature radius R=100 mm is chosen empirically in order to minimize additional bending loss. The interaction length is set fixed $L_{int}$ = 3 mm. First, directional coupler samples were inscribed without cladding structure modification and these samples showed no coupling between the waveguides even when the cores were brought together at the minimal possible distance. To surpass this problem several cladding tracks were skipped (see Figure 5a) to enable coupling between the neighbouring eigenmodes. In this setting the splitting ratio was controlled by modifying the distance between the waveguide core centers. Bringing the waveguides closer to each required removing more tracks in the interaction region. Table 1 summarizes the configurations and the corresponding splitting ratios of the fabricated couplers. The first value in splitting ratios defines the excited channel. The balanced splitting ratio which is of the most interest for most of the tasks [65,66] was found for d=14μm. The top and output facet view microscope images of the 2 × 2 directional coupler structures and the output optical intensive of the balanced coupler are depicted in Figure 5c–e. The average insertion loss per element is 7.8 dB.

**Figure 6:**
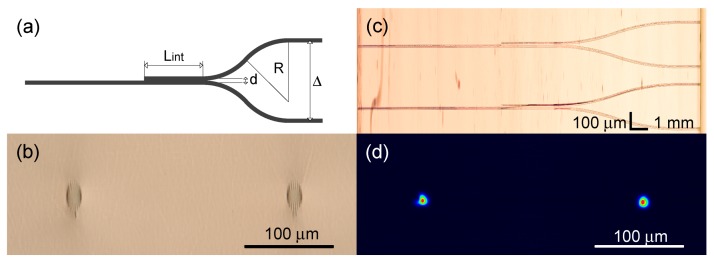
(**a**) The geometry of the 1 × 2 directional coupler. (**b**) The photograph of the cross-section of the output facet of the coupler. (**c**) The image of the 1 × 2 coupler. The image is stitched from the microscopy photographs with smaller field of view. (**d**) The image of the coupler output intensity distribution.

The 1 × 2 directional coupler has essentially the same structure as the 2 × 2 directional coupler except one of the input waveguides is removed and another is straightened before the interaction region (see Figure 6a). The balanced 49:51 splitting configuration remained unchanged compared to the configuration of the balanced 2 × 2 directional coupler. The top and output facet view microscope images of the 1 × 2 directional coupler structures and the output optical intensive of the balanced coupler are depicted in Figure 6b–d. The insertion loss per element decreased compared to the 1 × 2 directional coupler due to the bending region removing and estimated as 6.1 dB.

#### 3.2.2. 3 × 3 Directional Coupler

**Figure 7:**
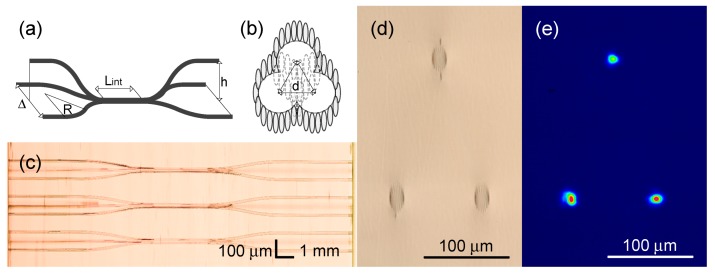
(**a**) The geometry of the 3 × 3 directional coupler structure. (**b**) The cross-section geometry of the interaction region. (**c**) The top-view image of the 3 × 3 couplers, composed by stitching microscope photographs with smaller field of view. (**d**) The microscope photograph of the end face of the 3 × 3 directional coupler and (**e**) the intensity distribution on the output of the structure with *d* = 14 μm.

We exploit 3D capabilities of the femtosecond laser writing method to fabricate 3 × 3 depressed cladding directional coupler. The 3 × 3 directional coupler consists of three waveguides coupled all together in the interaction region (see Figure 7a). At the input two of them lie in the same plane 250 μm under the surface of the sample on distance Δ= 100 μm. The third one starts at 70 μm depth and bends down to the interaction region approximately 240 μm below the surface, so h=180μm. The interaction length also is set fixed $L_{int}$ = 3 mm. The interaction region cross-section forms an equilateral triangle between the waveguide cores (see Figure 7b). The inner parts of the cladding structure of each waveguide were skipped to allow the coupling of three eigenmodes, 6 tracks from the first two waveguides each and eight tracks from the third. The splitting ratio is tuned by setting the appropriate distance between the centers of waveguide cores, details may be found in Table 2. The first value in splitting ratios defines the channel which was excited. The distance d=14.3μm corresponds to the balanced configuration of the 3 × 3 directional coupler. The top and output facet view microscope images of the 3 × 3 directional coupler structures and the output optical intensive of the 19:40:41 coupler are depicted in Figure 7c–e. The insertion loss per element was measured to be 8.6 dB.

## 4. Discussion and Conclusions

The main promising directions in the use of crystalline waveguides for implementing quantum memory and a quantum repeater on the photon echo are to complicate the topology of integrated optical circuits with active control elements. We have presented the technique to fabricate the basic elements of interferometric circuits, namely, 2 × 2, 1 × 2, 3 × 3 directional couplers in Tm${}^{3+}$:YAG crystal, which are suitable for functioning of a quantum memory cell. We provide the solution to the coupling problem of depressed cladding waveguides. Our solution might be exploited for creating an integrated platform that includes quantum states manipulation and storage processes on a single optical chip. Active control of the quantum state can be carried out in the future due to the electro–optical effect, since a common platform on a crystal with rare-earth impurities that transforms and stores quantum information is located in a helium cryostat, which does not allow using the thermo–optical effect.

## Figures and Tables

**Table 1:** The splitting ratio of the 2 × 2 directional coupler for different separation distance *d* between the waveguide core centers.

Separation Distance *d*, μm	Number of Skipped Tracks	Splitting Ratio, %
16.0	0	100:0
16.0	3	92:8
15.0	3	81:19
14.5	3	71:29
14.25	3	60:40
14.0	5	48:52

**Table 2:** The splitting ratio for 3 × 3 directional couplers with different distance *d* between waveguide core centers.

Distance between Core Centers *d*, μm	Splitting Ratio, %
14.0	19:40:41
14.1	22:39:39
14.2	25:38:37
14.3	33:34:33

## Abbreviations

The following abbreviations are used in this manuscript:

YAG	Yttrium aluminum garnet
TE	Transverse electric
TM	Transverse magnetic
AOM	Acousto-optical modulator
CMOS	Complementary Metal-Oxide-Semiconductor
